# Controlled Surface Textures of Elastomeric Polyurethane Janus Particles: A Comprehensive Review

**DOI:** 10.3390/polym16131835

**Published:** 2024-06-27

**Authors:** Ana Catarina Trindade

**Affiliations:** 1 CENIMAT|i3N, Department of Materials Science, School of Science and Technology, NOVA University of Lisbon, 2829-516 Caparica, Portugal; acrt@fct.unl.pt; 2 Atlântica, Instituto Universitário, Fábrica da Pólvora de Barcarena, 2730-036 Barcarena, Portugal

**Keywords:** Janus particles, elastomer, elastic instabilities, wrinkling, Janus fibers

## Abstract

Colloidal particle research has witnessed significant advancements in the past century, resulting in a plethora of studies, novel applications, and beneficial products. This review article presents a cost-effective and low-tech method for producing Janus elastomeric particles of varied geometries, including planar films, spherical particles, and cylindrical fibers, utilizing a single elastomeric material and easily accessible chemicals. Different surface textures are attained through strain application or solvent-induced swelling, featuring well-defined wavelengths ranging from sub-microns to millimeters and offering easy adjustability. Such versatility renders these particles potentially invaluable for medical applications, especially in bacterial adhesion studies. The coexistence of “young” regions (smooth, with a small surface area) and “old” regions (wrinkled, with a large surface area) within the same material opens up avenues for biomimetic materials endowed with additional functionalities; for example, a Janus micromanipulator where micro- or nano-sized objects are grasped and transported by an array of wrinkled particles, facilitating precise release at designated locations through wrinkle pattern adjustments. This article underscores the versatility and potential applications of Janus elastomeric particles while highlighting the intriguing prospects of biomimetic materials with controlled surface textures.

## 1. Asymmetric Janus Particles: A Short Overview

The remarkable progress achieved in the field of colloidal particles over the past century has led to numerous studies, innovative applications, and beneficial products. The term “colloid” originated from the Greek word “kolas”, meaning “the adhesive substance”, and was popularized by Thomas Graham, a chemist from Scotland, in 1870. Today, the science of colloids encompasses various branches of physical chemistry and technology [1].

Colloidal particles play a fundamental role in both natural phenomena and technological advancements. Colloids consist of particles with diameters ranging from hundreds of nanometers to a few micrometers, and they are uniformly dispersed in fluids. They are also known as colloidal dispersions because the particles remain dispersed in the fluid, not settling at the bottom, as happens in a suspension [2]. Colloidal systems are omnipresent in our daily lives, with a history that dates back to ancient times. They exert a significant impact and hold substantial influence, finding extensive applications in various goods-manufacturing processes (including the production of potable water), biotechnological separation processes, wastewater treatment, and even orthomolecular therapy. Numerous tangible examples highlight their utility, such as mayonnaise, tinted glass, milk, butter, turbid water, gelatin, blood, hair spray, clouds, and paper. Conversely, certain colloids can be undesirable, such as soot or particle pollution. From a scientific perspective, colloids present a captivating and intriguing nature as they exhibit characteristics that combine the essence of both microscopic particles and macroscopic objects. Their size allows them to be influenced by thermal energy while exhibiting Brownian diffusion slow enough to be observed in real time through optical microscopy. The behavior of colloidal systems is governed by the dominant interactions, such as electrostatic, van der Waals, and dipolar forces, mirroring the behavior observed in atomic and molecular systems. If successfully assembled into functional structures, colloidal nanoparticles have the potential to become the building blocks of future materials, comparable to “atoms” and “molecules” [3].

The objective of this review is to provide a comprehensive overview of the advancements in the field of Janus particles, with a specific emphasis on the controlled surface textures of elastomeric polyurethane Janus particles. We aim to highlight the significance of these materials in various applications, including their unique structural characteristics, synthesis methods, and potential functionalities. This review will cover the following key aspects: historical development and fundamental concepts of Janus particles; detailed examination of surface texture control in elastomeric polyurethane Janus particles; advances in fabrication techniques and material properties; and application-specific discussions, particularly in fields such as biomedical engineering, environmental science, and materials science. By systematically exploring these topics, this review seeks to provide a valuable resource for researchers and to stimulate further innovation in this evolving area of study.

Janus particles, a unique category of colloidal particles, were first introduced in a scientific publication in 1985 by Lee et al. [4]. The study focused on asymmetric poly(styrene)/poly(methyl methacrylate) (PMMA) lattices produced through seeded emulsion polymerization [4]. Subsequently, Casagrande and Veyssié conducted further investigations using glass spheres and developed a method for creating hydrophobic nanobeads with one hemisphere treated with octadecyl trichlorosilane, while the other hemisphere was protected with a cellulosic varnish [5]. However, it was not until 1991 that the concept of Janus particles gained significant attention when de Gennes mentioned them in his Nobel Lecture [5]. De Gennes proposed the idea of amphiphilic colloidal particles with one polar and one nonpolar face, functioning as a new type of surfactant [5,6]. These particles would adsorb at liquid interfaces and exhibit amphiphilicity, stabilizing the interface while allowing for “breathing” through interstices between the Janus particles. Since then, substantial progress has been achieved in utilizing this anisotropy for materials design and assembly.

In ancient mythology, Janus, the god with two faces, symbolized duality. Traditionally, statues of Janus were placed at temple entrances, positioned to face in opposite directions simultaneously. Similarly, Janus asymmetry can be generally defined as asymmetric particles with at least two surface regions or bulk compositions differing in their physicochemical properties [6]. These structures exist on a micro- or nanoscale and exhibit non-centrosymmetric architectures. Janus particles, a special subset of colloidal particles, feature different chemical compositions on their two hemispheres. The term “Janus” derives from their unique architectural characteristic of having two surfaces or sides with contrasting chemistries and/or polarities. The most prevalent form of Janus particle is a nanosized sphere with hemispherical amphiphilicity, meaning that one hemisphere is polar while the other hemisphere is nonpolar [5].

**Figure 1:**
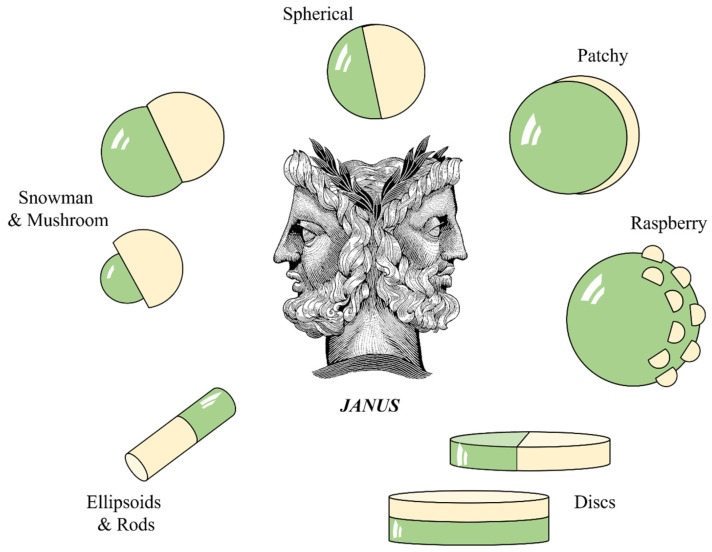
Types of Janus particles: ellipsoids and rods; snowman and mushroom; spherical; patchy; raspberry; and discs.

In the realm of modern materials research, it is common to observe parallels between Janus particles and natural entities like fungi, plants, and viruses [7]. While the concept of Janus particles initially garnered limited attention, it has now become widespread in the field of colloidal systems [8,9,10,11]. Over the past 20 years, the original Janus concept has captivated scientists and engineers, leading to the exploration of Janus particles with different shapes. These shapes include perfectly spherical particles with two hemispheres with different surface properties, as well as more commonly observed forms such as snowman or dumbbell shapes (Figure 1). Janus particles can be prepared in a wide range of sizes, and different bulk or surface properties can be “loaded” through selective modification on each of the Janus lobes, sometimes combining in surprising ways. Researchers have employed various materials, including inorganic substances [12,13,14,15,16,17,18], dendrimers [19,20,21,22,23,24,25,26,27,28], liquid crystalline compounds [23,24,29,30,31,32,33,34,35,36,37], and polymers [38,39,40,41,42,43,44,45,46,47,48,49,50], to synthesize asymmetric architectures resembling Janus particles.

## 2. Janus Particles: General Synthetic Routes and Applications

Since the first successful creation of Janus particles in 1988 [51], numerous innovative methods have emerged for fabricating and developing these structures. These approaches cater to both academic research purposes and large-scale industrial applications.

Various powerful tools have been employed to engineer the production of specific target structures within Janus particles. These tools include chemical methods like selective crystallization and deposition; physical methods like electrified jetting, microcontact printing, emulsion drying, selective deposition, surface templating, direct writing, and lithography; as well as biologically inspired methods utilizing plant extracts, fungi, or viruses to synthesize metal nanoparticles of different shapes. These novel approaches to particle synthesis offer a wide range of particle anisotropies with diverse and exotic structures, leading to unique properties such as electronic and optical characteristics.

The techniques used for synthesizing Janus particles can be broadly classified into two categories: surface modification (or masking and asymmetric modification) and compartmentalization. Surface modification involves selectively altering one part of isotropic particles to create an anisotropic surface. 

The masking and asymmetric modification method is commonly used nowadays [12,13], as it capitalizes on the well-established knowledge of synthesizing nano- and colloidal particles with isotropic surface chemistry. By protecting a portion of the initially uniform particle during the modification process, Janus particles can be easily derived. Masks, templates, geometric constraints, and physical vapor or lithography techniques can be employed to achieve this [14,15,16,17,18]. An alternative surface modification method involves physical deposition using electron beam evaporation or sputter coating [18,19,20,21,22,23,24], allowing for the design of Janus particles with elaborate geometries. Directional coating with electron beam evaporation on a monolayer of colloidal particles enables the production of anisotropic particles coated with metals or other materials in precise half–half geometry [25,26,27,28]. By applying sequential physical deposition and/or new masks, Janus anisotropic particles with more than one patch can also be obtained. Coating can also enable the surface modification of colloidal particles, especially if particular surface chemistry properties are desired.

This enables the creation of Janus particles that exhibit anisotropy not only on their surfaces but also in their bulk [29]. Depending on the chosen synthesis technique, a diverse range of distinct compartments can be achieved, thereby expanding the potential functions and applications of these Janus particles.

Various methods have been tested and developed to synthesize Janus particles with multiple separated compartments. These methods include microfluidics [30,31,32,33,34,35], electrodynamic co-jetting [36,37,38], chemical synthesis and polymer self-assembly [18,39,40,41,42,43,44].

Beyond the synthesis aspect, the appeal of the Janus concept extends to two isotropic particles, depending not only on the particle separation but also on the mutual concentration and relative orientations. Due to their distinct faces, Janus particles respond differently to external fields such as electric, magnetic, chemical gradients, or temperature gradients, leading to the assembly of micelles and membranes crucial for various biological functions. The versatility of Janus particles enables the fine-tuning of the properties and functions in self-assembled materials, providing innovative approaches for designing new materials with controlled and desirable characteristics. 

The unique feature of possessing two distinguishable faces in Janus anisotropic particles opens up numerous fascinating potential applications. These particles can serve not only as solid surfactants, as initially proposed by de Gennes [5], but also find use in various other novel applications. Some of these applications include acting as interfacial stabilizers [45,46,47,48,49,50], microprobes and biosensors [52,53,54,55,56], drug delivery vehicles [57,58,59,60,61], components for reconfigurable materials like electronic paper or displays [62,63,64], nano engines [65,66,67,68,69], and micromotors [70,71,72,73]. Janus particles also play significant roles in optical imaging [74,75,76,77], diagnostic applications [78,79,80], and catalysis [81,82,83,84]. 

The broad definition of Janus particles encompasses micro- and nanosized particles with anisotropic properties, possessing two regions of different surface chemical compositions. The interactions among these particles are influenced not just by their separation but also by their relative orientation [10,85]. As a result, many micro- and nanoscale particles exhibiting such anisotropic characteristics are categorized as Janus particles. 

## 3. Surface Periodic Patterns: Wrinkling and Buckling

**Figure 2:**
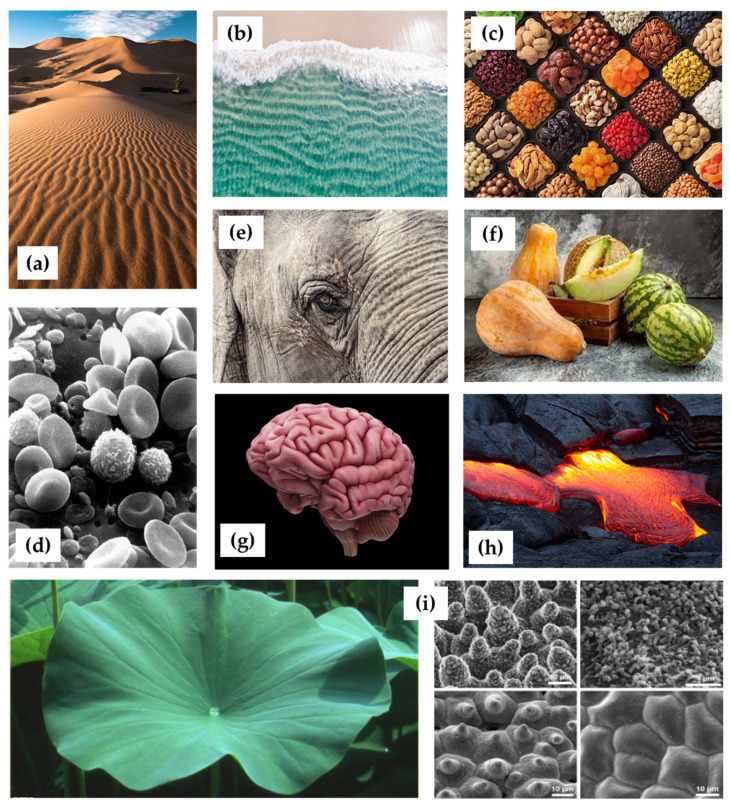
Examples of the periodic patterns widespread in nature: (**a**) patterns in the sand dunes of a desert [88]; (**b**) waves’ regular pattern [89]; (**c**) wrinkles in dehydrated fruits [90]; (**d**) blood cells and irregularly shaped leukocytes [91]; (**e**) an elephant’s wrinkly skin, where the wrinkles are actually a complex web of “microvalleys” formed as the outermost skin layer (“epidermis”) thickens and bends with age, causing it to crack [92]; (**f**) wrinkled patterns observed in pumpkins and melons [93]; (**g**) surface patterns of brain folding [94]; (**h**) photograph of a lava sheet buckling and deforming under the compressional stress [95]; and (**i**) photo of lotus leaves, which exhibit extraordinary water repellency on their upper side, and scanning electron microscopy (SEM) image of the upper leaf side, showing the hierarchical surface structure consisting of papillae, wax clusters and wax tubules [96].

Periodic patterns, both static and dynamic, are widespread in nature (see Figure 2), appearing in various sizes and shapes, such as plant pollens and peppercorn seeds. From small wrinkles on soft materials like skin to larger wavelength buckles seen in mud cracks, sand dunes, waves in water or lava flows, these wrinkled patterns can be observed in pumpkins, melons, nuts, dehydrated fruits (such as grapes and prunes), and the skin of large animals like elephants, rhinos, and reptiles. 

Biological systems also exhibit numerous self-organized periodic patterns [86,87], such as the striped patterns in intestines and arteries, the surface patterns of the brain folds, and the microtopographical surface structures on lotus leaves and petals. Even at the cellular level, latitudinal undulations have been discovered in human neutrophil cells, mineralized yeast cells, fish spawns, and venom-infected sperm. Many of these intricate patterns have evolved through natural selection over millions of years, contributing to the survival and prosperity of species in their respective environments. These natural designs offer advantages, such as reducing the turbulent drag and hydrodynamic friction in sharks and dragonflies, exhibiting special wetting and cleaning properties as seen in the lotus effect, and minimizing friction and abrasion for animals like the “sandfish” that can “swim” under the sand. Wrinkles and folds also play a critical role in facilitating nutrient absorption in intestines. 

These examples demonstrate how wrinkled patterns arise in nature through various physical and biological processes, and how they are invaluable in efforts to improve or mimic nature, such as aiding cell adhesion at the interfaces of biomaterials and biological systems. The formation of these patterns is often a result of the interplay between external forces, material properties, and growth dynamics. The study of such patterns can offer insights into the underlying processes and provide inspiration for innovative design and engineering solutions. Due to the myriad benefits and diverse applications of these periodic structures, researchers have developed a vast array of fabrication approaches in recent years.

## 4. Controlling Surface Textures of Polyurethane Elastomeric Janus Particles

Harnessing the capability of elastomers to alter their surface through buckling and wrinkling, a simple, cost-effective, and accessible method has been developed for fabricating Janus elastomeric particles with varied geometries (including planar films, spherical particles, and cylindrical fibers) from a single elastomeric material using readily available chemicals.

### 4.1. Wrinkling in Free-Standing Flexible Elastomers

In this subsection, we will examine various surface topographies of elastomeric particles. The predominant focus in this field has been on polydimethylsiloxane (PDMS) elastomers [97,98,99]. However, we will concentrate on polyurethane/urea elastomers due to our expertise in this area and also to their role as precursor materials for the wrinkled labyrinthic Janus particles discussed herein. Nevertheless, similar effects can be qualitatively achieved with any other network-forming polymers.

**Figure 3:**
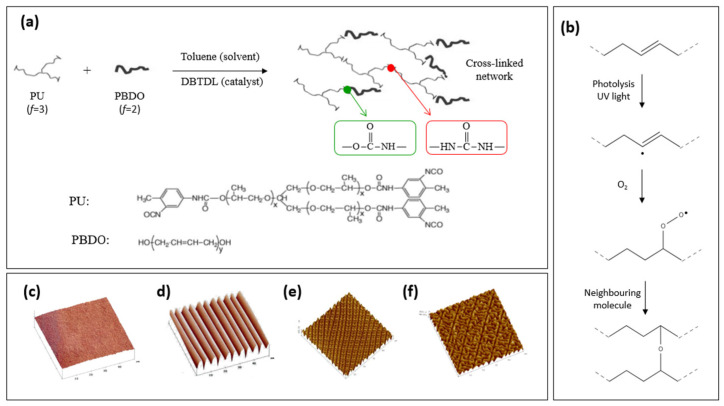
(**a**) Synthesis of bi-soft segment urethane/urea elastomers from poly(propylene oxide) (PU), with a degree of polymerization (DP) equal to 20 and a functionality *f* = 3 and polybutadiene diol (PBDO with DP = 50 and *f* = 2). (**b**) Schematic of PBDO double bond opening by UV in the presence of oxygen, which promotes the cross-linking reaction on the irradiated surface of the spheres. (**c**–**f**) AFM images of a polyurethane/polybutadiene diol (PU/PBDO) film: (**c**) immediately after UV irradiation and before any mechanical stress has been applied, the film is smooth; (**d**) stretching the film along one direction induces a regular one-dimensional wrinkling; (**e**) further stretching along a second direction perpendicular to the first induces a two-dimensional texture resulting from the superposition of the two one-dimensional waves of wrinkling; and (**f**) swelling the film in toluene imprints the texture permanently. (Reproduced with permission from ref. [102] Copyright © 2006 Elsevier; and reproduced with permission from ref. [100] Copyright © 2007, EDP Sciences, Società Italiana di Fisica and Springer-Verlag).

In block copolymers that combine rigid and flexible segments, when one or more of the reactive segments possesses multiple functional groups (functionality, *f* > 2), it enables chain cross-linking, leading to the formation of a polymeric network. Earlier studies involved the preparation of polyurethane/urea elastomeric membranes [100,101] from precursor solutions comprising a polyurethane prepolymer (PU, *f* = 3) and hydroxyl-terminated prepolymer (PBDO, *f* = 2). These membranes displayed remarkable behavior during stretching. Unlike typical materials, which turn whitish upon stretching and then revert to clarity upon relaxation, these films remained relatively clear during stretching and turned translucent upon release. This intriguing mechanic-optical phenomenon was reversible and contingent upon the preparation conditions and applied stress. Microscopic examination revealed distinct surface characteristics, featuring bands and wrinkles oriented perpendicular to the stretching direction. Moreover, it was discovered that the surface morphology of free-standing urethane/urea elastomeric films could be systematically adjusted in a reproducible and reversible manner. Furthermore, six distinct pattern states were identified for the same elastomeric material, elucidating their relationships and manipulative potential (see Figure 3).

**Figure 4:**
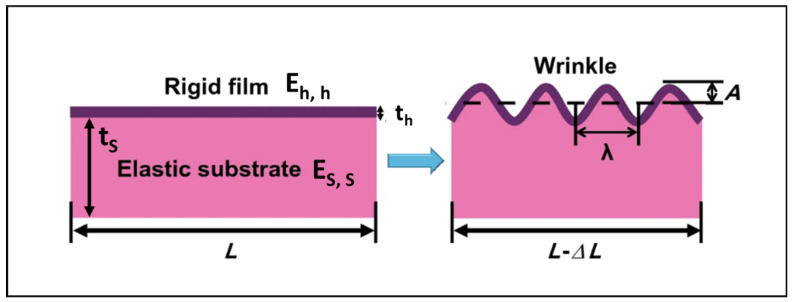
Wrinkle formation through buckling instabilities in various bilayer systems. Schematic for wrinkle formation in a “bilayer system”. E and t are the film elastic modulus and film thickness, respectively. The subscripts “h” and “s” denote the top (stiff) layer and bottom (soft) layer; λ is the wrinkle wavelength and A is its amplitude. (Adapted from ref. [103] under the terms of under the Creative Commons CC BY license (https://creativecommons.org/licenses/by/4.0/).

The mechanism underlying the formation of wrinkled patterns on elastomeric films can be elucidated through the model proposed by Bowden in 1998. In this model, the film is conceptualized as comprising two distinct regions: a rigid skin atop a softer bulk. In the case of urethane/urea films, the rigid skin results from the enhanced cross-linking of the polyurethane induced by ultraviolet (UV) irradiation. When the film undergoes stretching, the skin and substrate deform differentially: the rigid skin experiences more pronounced plastic deformation than the substrate. Upon stress removal, while the substrate seeks to return to its original dimensions, the skin remains expanded. Consequently, a net compressive stress acts on the skin (due to the bulk) and a net tensile stress acts on the bulk (due to the skin) (see Figure 4).

The skin operates akin to an elastic plate under in-plane compression, inducing buckling. The wavelength of the wrinkles, denoted as λ, arises from the interaction between two factors: the bending energy of the thin, rigid skin (preferring shorter wavelengths) and the bulk elastic energy of the soft core (favoring longer wavelengths). Consequently, the flat state transitions into a wrinkled state with a wavelength determined by [103]:

λ ~ t_h_ (E_h_/E_s_)^1/3^ (1)

where E_h_ and E_s_ represent the Young’s moduli of the rigid skin and the flexible core, respectively, and t_h_ denotes the thickness of the rigid skin, presumed to be significantly smaller than that of the substrate. Consequently, the wavelength of the wrinkle’s scales changes with the thickness of the rigid layer, which stands as the sole pertinent length scale in this scenario.

Various surface textures were induced in free-standing flexible polyurethane elastomers through applied strains or solvent-induced swelling, exhibiting well-defined wavelengths ranging from the sub-micron to millimeter scales, easily adjustable, and thus deemed relevant for potential medical applications, particularly in bacterial adhesion studies. Preliminary findings on *Staphylococcus epidermidis* adhesion to modified polyurethane/urea elastomeric samples have already been reported [104], highlighting the utility of these elastomers as model surfaces in bacterial adhesion research.

### 4.2. Janus Wrinkled Spheres

Alternative Janus membranes and particles with varying geometries may offer greater convenience for potential applications. Wrinkled spheres and colloids (comprising PDMS, silica, or silicone rubbers) have been synthesized through diverse methods, including direct replication from wrinkled templates [105], directional UV-induced reactions [106], selective contact thermodynamic reactions [107], stress-driven mechanisms [108], photolithography [109], aerodynamic dry control [110], Pickering emulsion [111], selective O_2_ plasma exposure [112], chemical oxidation [113], or via protective masking techniques [114], among others. Despite the extensive research in this area, the exploitation of wrinkling labyrinth patterns on elastomeric Janus particles has primarily been limited to spheres derived from polyurethane/urea systems, as mentioned earlier.

**Figure 5:**
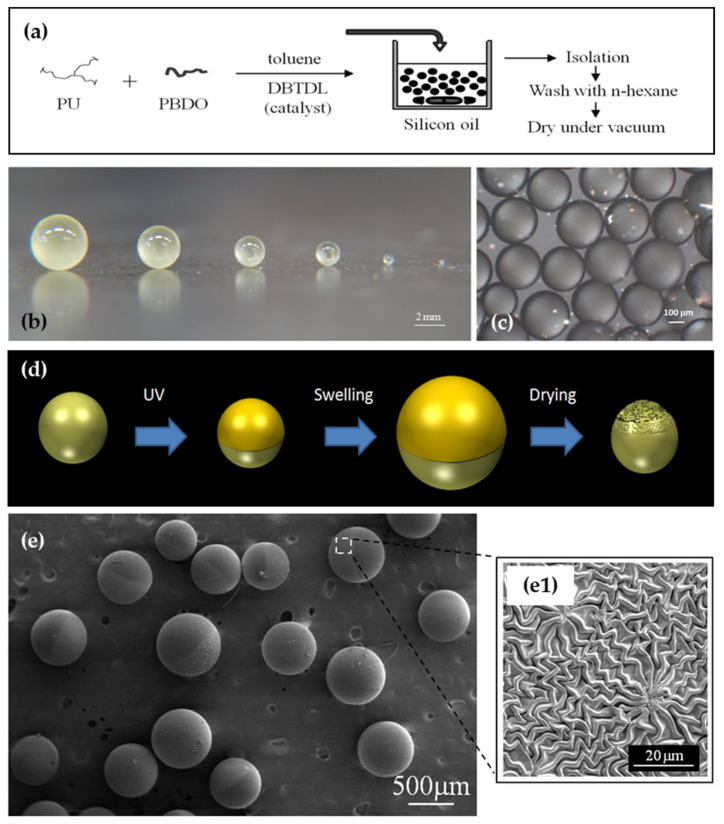
(**a**) Scheme of the simple fluidic procedure to produce monodisperse PU/PBDO spheres. (**b**) Monodisperse milli- and micro-sized spheres can be produced using a simple fluidic device. (**c**) POM images of spheres with a diameter of about 300 µm. (**d**) Schematic of the production of asymmetric elastomeric particles with wrinkling labyrinth patterns on one hemisphere: UV irradiation is followed by swelling the particles in toluene and drying them. (**e**) Photos of the spheres’ scattering wrinkled and shiny smooth hemispheres after UV irradiation. ((**e1**) shows the detail of the asymmetrically wrinkled top surface of a sphere) (Adapted with permission from ref. [116] © 2011, American Chemical Society.; and Adapted from ref. [117] with permission from the Royal Society of Chemistry).

Utilizing the same chemical principles employed for films, a straightforward microfluidic device (adapted from ref. [115]) can be utilized to fabricate spherical elastomeric polyurethane/urea particles spanning from tenths of a micron to a few millimeters in diameter. Following production, washing, and isolation, one hemisphere of these spheres can be selectively modified while preserving the surface of the other hemisphere. This is achieved by temporarily masking the intended unmodified hemisphere during UV irradiation, typically accomplished by depositing the precursor particles onto a solid opaque cellulosic film. Upon swelling and subsequent de-swelling of the entire sphere in an appropriate solvent, the wrinkled surface characteristic of Janus spheres will emerge exclusively on the irradiated hemisphere. Hence, akin to elastomeric urethane/urea films, these Janus spheres may exhibit a wrinkled hemisphere alongside a smooth counterpart (see Figure 5).

During swelling in a suitable solvent, both the inner (softer) core and the outer (stiffer) skin undergo deformation to the same extent, resulting in a smooth outer surface. Upon drying (de-swelling), the entire elastomer network contracts due to solvent evaporation and the loss of unreacted prepolymer blocks (referred to as “sol fraction”). The softer core, with its lower cross-linking density, returns to its original dimensions, while the densely cross-linked stiff skin does not. This discrepancy in size generates internal stress, prompting the buckling instability of the skin and giving rise to an asymmetric Janus elastomeric sphere with distinct smooth and wrinkled hemispheres.

**Figure 6:**
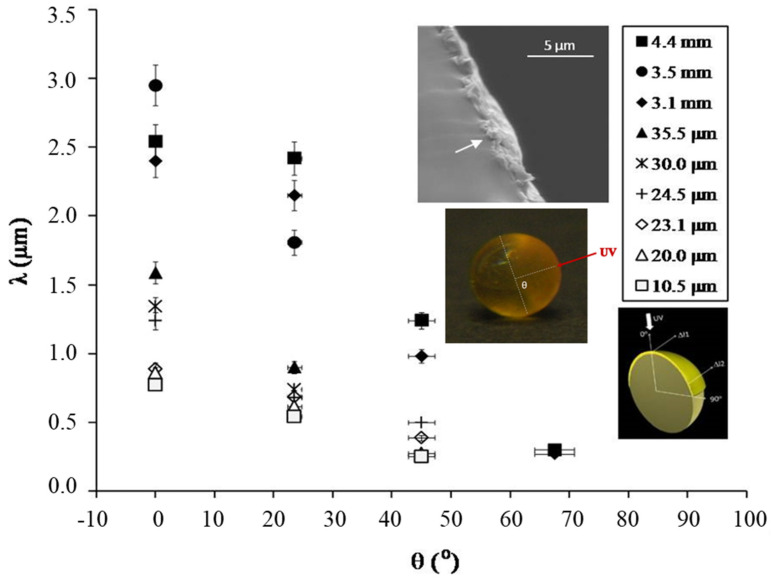
Dependence of λ, the wrinkle wavelength of linear instability, on the skin thickness and sphere diameter. (Adapted with permission from ref. [116]. © 2011, American Chemical Society).

Considering the model proposed for films [118], in these elastomeric spheres, the wavelength λ of the buckling linear instability relies on the dimensionless ratio of the outer skin thickness (t_h_) to the sphere’s radius (R). The relationship between the wrinkle wavelength λ and the co-latitude θ (where θ = 0° corresponds to the point where the incident UV light was shone perpendicular to the sphere, and θ = 90° corresponds to the boundary between the two half-hemispheres) is illustrated in Figure 6. The thickness of the stiff skin (depicted in the inset) diminishes from θ = 0° to θ = 90°, distinctly influencing λ. For cylindrical geometries, a generalized version of Equation (1) was derived through analytical methods:

λ/R ~ (t_h_/R)^3/4^ (2)

Applicable for R/h < 50 (beyond this threshold, Equation (1) is essentially restored), a fitting of our experimental data (pertaining to Janus spheres) yielded an exponent of 0.82 instead [116]. This finding aligns reasonably well with the numerical findings of Cao et al. [119] for micron-sized particles featuring a sealed silica shell on a silver substrate, indicating an exponent close to 0.8.

### 4.3. Janus Wrinkled Fibers

Employing the same PU/PBDO system used for films and spheres, Trindade et al. investigated the development of wrinkling instability in long cylindrical elastomeric fibers with nano/micron-scale radii [120]. These fibers are synthesized through a two-step chemical process, adapted from the previously described procedure and details: first, the reaction between PU and PBDO; followed by a second step induced by UV irradiation.

PU/PBDO fibers were fabricated using the electrospinning technique, which enables the continuous production of fibers collected onto a designated target. The term “electrospinning” derives from its reliance on an electric field for fiber production, where fiber deposition is driven by the electrostatic repulsion of electric charges.

The typical configuration of an electrospinning deposition system includes a syringe containing the polymer solution intended for fiber production, a diffuser pump regulating the solution flow rate through the syringe needle, a metallic target where deposited fibers are collected and maintained at zero electrical potential, and a voltage source to establish a potential difference between the needle tip and the target. A rotating target positioned at a distance from the syringe was utilized in our setup. As the viscous elastomeric solution exits the needle, it is propelled toward the target by the applied voltage, forming long, slender fibers.

**Figure 7:**
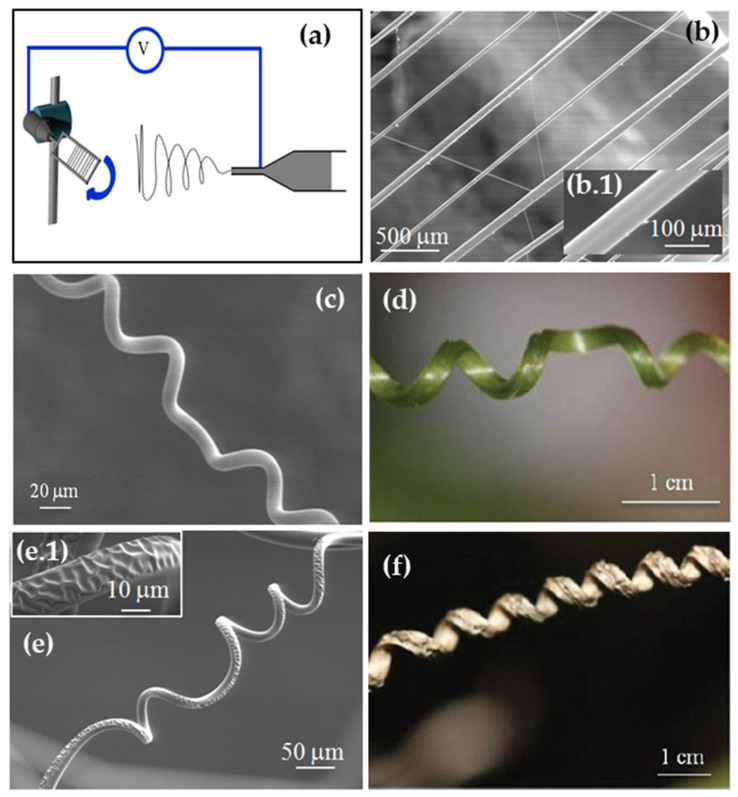
(**a**) Schematics of fiber production by the electrospinning technique: syringe filled with elastomer PU/PBDO precursor solution and syringe needle, connected to high-voltage supply; elastomeric fiber accelerating toward a rotating target (used to obtain aligned and tautly suspended fibers). (**b**) SEM image of aligned fibers as electrospun, with a smooth surface (see inset (**b.1**)). (**c**,**d**) Electrospun PU/PBDO fibers mimicking young and old plant tendrils: a smooth fiber (**c**) mimics a young tendril (**d**); an asymmetrically wrinkled fiber (**e**) mimics an old tendril (**f**). ((**e.1**) shows the detail of an asymmetrically wrinkled fiber in (**e**). Notice the wrinkling is on the outside of the helix, consistent with the assumption that the stiff skin contracts less than the soft core on drying). (Adapted from ref. [120], © WILEY-VCH Verlag GmbH & Co. KGaA, Weinheim, reproduced with permission).

A schematic representation of the fabrication process is depicted in Figure 7. Due to the geometry of the target, the fibers remained taut throughout the process. 

After being collected onto the target and dried, the fibers undergo UV irradiation on only one side and are swollen in toluene while still attached to the target and under tension. At this stage, the fibers are smooth on both sides, albeit exhibiting anisotropy: the irradiated side features a stiff skin, whereas the non-irradiated side possesses a soft skin. This anisotropic behavior, coupled with solvent evaporation, results in a size disparity between the skin and the core. Upon removal from the target, the tension is released, causing the fibers to immediately coil into helices. However, the stiff skin remains smooth, and the wrinkled surface only becomes apparent after the fiber assumes a well-defined helical shape [120].

Trindade et al. proposed [120] a simple elastic model, predicting that as the fibers de-swell, they initially coil while remaining smooth. It is only at a critical curvature value that the fibers cease coiling and wrinkles emerge on the irradiated surface. As de-swelling progresses, these wrinkles increase in amplitude while maintaining a distinct wavelength. Once again, akin to the prediction for spheres, both coiling and wrinkling are governed by the interaction between bending the skin and dilating the core. The elastic energy of the coiled, non-wrinkled state is proportional to the square of the skin–core size mismatch (ε^2^), whereas the elastic energy of the wrinkled state increases linearly with ε. As the fiber dries, ε increases, causing the smooth fiber to coil with a radius proportional to ε − 1. Only at a critical mismatch, ε_c_ ~ (E_SC_/E_SS_)^2/3^, does it become energetically favorable to cease coiling and initiate wrinkling, with a wavelength determined by Equation (1).

The same qualitative behavior can be observed, albeit on a vastly different length scale, in the tendrils of climbing plants as they age and dry: initially, they coil, then they wrinkle (see Figure 7).

Recent advancements in colloid research have facilitated the synthesis of Janus particles through a multitude of techniques, allowing for the production of substantial quantities of well-defined Janus particles with diverse architectures. Furthermore, certain preparation methods that have been developed can be scaled up to industrial levels, paving the way for feasible technological applications of Janus particles. As novel methods for Janus particle synthesis continue to emerge, potential applications are beginning to surface, particularly in the realms of highly specific sensors, self-propelled particles, and interface stabilization. However, the current high production costs associated with Janus particles may impose limitations on certain technological applications.

## 5. Applications and Future Perspectives 

**Figure 8:**
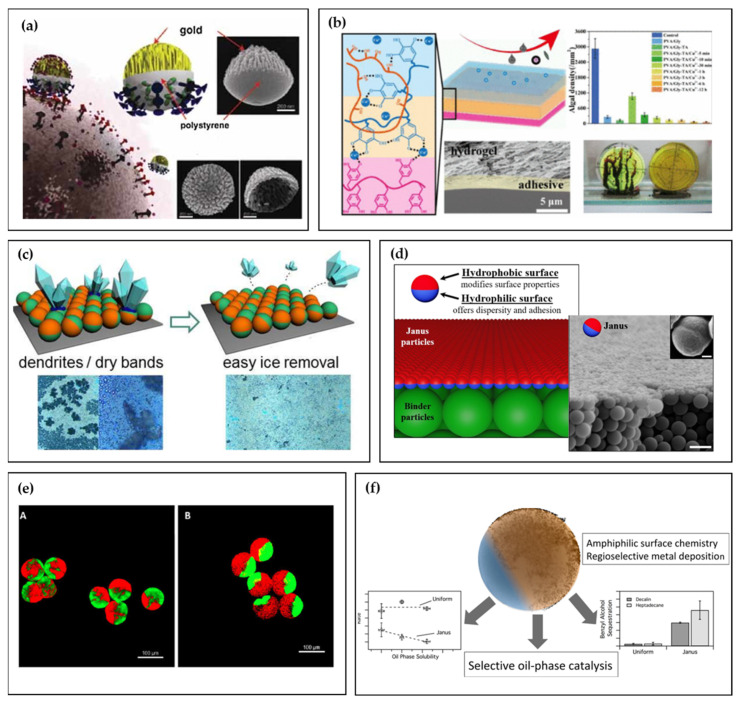
(**a**) Nanocorals in the Janus structure as multifunctional targeting, sensing, and drug delivery nanoprobe; inset: scanning electron microscope (SEM) images of Janus-structured nanocoral probes. Reproduced with permission from ref. [78] (2010), © Wiley, 2010. (**b**) Transparent Janus hydrogel wet adhesive for underwater self-cleaning (Adapted from ref. [133] Copyright © 2021 American Chemical Society). (**c**) Functional surfaces with effective anti-icing and de-icing capability based on hybrid Janus particles (adapted from ref. [126] Copyright © 2016, American Chemical Society). (**d**) Amphiphilic Janus particles are mixed with homogeneous binder particles with strong adhesion to create robust hydrophobic coatings through a self-stratification process (Adapted from ref. [134] licensed under a Creative Commons Attribution-NonCommercial 3.0 Unported Licence). (**e**) Fluorescence confocal microscopy images for Bodipy-pectin homo Janus (**A**) and hetero Janus (**B**) hydrogel microparticles with dluoresceinamine-coupled pectin and alginate, respectively (Adapted with permission from ref. [130] @ 2022 John Wiley and Sons). (**f**) Amphiphilic Janus particles with a catalyst selectively loaded on either the hydrophobic or hydrophilic region are promising candidates for efficient and phase-selective interfacial catalysis (Adapted with permission from ref. [131]. Copyright 2020 American Chemical Society).

The controlled surface textures of elastomeric Janus particles have emerged as a fascinating area of research with promising applications across a multitude of disciplines (see Figure 8). This comprehensive review aims to delve into the myriad applications enabled by the unique properties of these particles, offering insights into their potential impact on various fields. The results obtained in relation to PU/PBDO elastomeric Janus membranes, particles, and fibers discovery open up new avenues in biomimetic materials, where functionalities can be introduced by incorporating “young” (smooth, small-surface area) and “old” (wrinkled, large-surface area) regions within the same material; for instance, a Janus micromanipulator constructed using an array of wrinkled particles to grasp and transport micro- or nano-sized objects, releasing them precisely by adjusting the pattern.

In the realm of biomedicine, elastomeric Janus particles with controlled surface textures hold tremendous potential for advancing drug delivery systems. Recent studies have demonstrated their ability to facilitate controlled and targeted drug release, thus overcoming many of the limitations associated with conventional drug delivery methods [121]. Furthermore, these particles have shown promise in enhancing the biocompatibility of biomaterials used in implants and prosthetics, paving the way for the development of next-generation biomedical devices [122].

In materials science, the versatility of elastomeric Janus particles with controlled surface textures has sparked considerable interest in the development of smart materials. By exploiting their responsive surface properties, researchers have successfully created self-cleaning surfaces capable of repelling contaminants and maintaining cleanliness in various environments [123]. Additionally, these particles have been utilized in the fabrication of responsive actuators and sensors, enabling the development of intelligent materials capable of adapting to changes in their surroundings [124].

At the micro/nanoscale, elastomeric Janus particles have found applications in microfluidics, where their precisely controlled surface textures have been leveraged to manipulate the fluid flow and enhance the performance of microfluidic devices [125]. Moreover, recent advancements have demonstrated their potential in energy-harvesting technologies, where changes in the surface textures can be harnessed to generate mechanical energy for various applications, including powering small-scale electronic devices [126,127].

In the field of coating and adhesion technology, elastomeric Janus particles with controlled surface textures offer innovative solutions for enhancing surface properties and controlling adhesion in diverse applications. Recent studies have shown their efficacy in creating anti-adhesive coatings capable of repelling liquids and preventing fouling on surfaces [64]. Furthermore, these particles have been employed to develop novel printing and packaging materials with controlled adhesion properties, enabling precise control over adhesion in printing and packaging processes [128,129,130].

Beyond traditional applications, elastomeric Janus particles with controlled surface textures are also finding utility in catalysis and phase-separation processes. Recent research has demonstrated their potential as selective catalysts, facilitating specific chemical reactions with high efficiency and selectivity [131]. Additionally, these particles have been utilized in phase-separation processes to achieve the efficient separation of immiscible components, thereby enabling the purification and recycling of materials [132].

Future research efforts will likely focus on further refining the control over the surface textures and expanding the range of functional modifications available. This will involve developing new synthesis methods and advanced characterization techniques to better understand and manipulate the surface properties of Janus particles. Interdisciplinary collaborations will be crucial in these endeavors, bringing together expertise from materials science, chemistry, biology, and engineering to address complex challenges and explore new applications.

Advancements in computational modeling and machine learning could also play a significant role in predicting and optimizing the behavior of Janus particles, accelerating the development of new applications. By integrating experimental and theoretical approaches, researchers can more effectively design particles with tailored properties for specific applications.

In conclusion, the diverse applications of the controlled surface textures of elastomeric Janus particles underscore their significant potential to drive innovation across various fields. Continued research in this area holds promise for the development of novel technologies with broad-ranging societal impact.

## 6. Challenges and Limitations

The field of elastomeric polyurethane Janus particles has witnessed significant advancements, yet several challenges and limitations remain that must be addressed to enable further progress and broader application.

One of the primary limitations is the difficulty in scaling up current synthesis methods while maintaining the reproducibility. Most synthesis techniques are optimized for laboratory-scale production and translating these methods to industrial-scale production poses significant challenges. Issues such as the batch-to-batch variability and the need for precise control over the reaction conditions make large-scale production difficult. Ensuring uniformity and consistency in particle production at an industrial scale is essential for practical applications, yet achieving this remains a significant hurdle.

Achieving precise control over the surface textures and morphology of Janus particles is another critical challenge. The complex nature of the synthesis process, which often involves multiple steps and varying conditions, can lead to inconsistencies in the particle size, shape, and surface properties. This variability can affect the performance of the particles in their intended applications. Advanced fabrication techniques and a better understanding of the underlying mechanisms are needed to improve control over these parameters. Research into novel synthesis methods that allow for more consistent and precise control over the particle morphology and surface texture is crucial.

The long-term stability and performance of Janus particles in various applications are also areas of concern. Factors such as the environmental conditions, chemical stability, and mechanical durability can significantly affect the functionality of Janus particles over time. For instance, exposure to varying pH levels, temperatures, and other environmental factors can degrade the materials, compromising their effectiveness. Research into enhancing the stability and performance of these particles is essential for their practical application in real-world scenarios. Developing coatings or modifications that can protect the particles from environmental degradation could be one approach to addressing this issue.

Understanding the structure–property relationships of Janus particles is crucial for optimizing their design and functionality. However, accurately characterizing these particles poses significant challenges due to their complex and often heterogeneous nature. Advanced characterization techniques and computational models are needed to better understand how the structure of Janus particles influences their properties and performance. For instance, how the distribution of different materials on the particle surface affects their interaction with the environment and their overall behavior. This understanding is vital for designing particles with specific properties tailored for particular applications.

Moreover, the current challenges in characterizing these particles often stem from their nanoscale dimensions and the intricate nature of their surface properties. Techniques such as electron microscopy, atomic force microscopy, and various spectroscopic methods are required to probe these features in detail. However, these techniques can be time-consuming and may not always provide a complete picture of the particle’s properties. Developing more efficient and comprehensive characterization methods will be critical for advancing the field.

Additionally, addressing the challenges related to the application of Janus particles is vital. For instance, in biomedical applications, ensuring biocompatibility and minimizing potential toxicity are crucial. In environmental applications, the impact of these particles on ecosystems must be thoroughly understood and mitigated. Researchers must also consider the economic and environmental aspects of large-scale production and application, ensuring that the benefits of Janus particles are realized without undue negative consequences.

Addressing these challenges is critical for the continued development and application of elastomeric polyurethane Janus particles. Future research should focus on developing scalable synthesis methods, enhancing control over particle morphology, improving long-term stability, and advancing characterization techniques. By overcoming these obstacles, the full potential of these innovative materials can be realized, paving the way for their use in a wide range of applications, from biomedical devices to advanced materials and environmental remediation.

## 7. Conclusions

This review highlights the significant advancements in the field of Janus particles with controlled surface textures, particularly those composed of elastomeric polyurethane. The key contributions of this review include a detailed analysis of the synthesis techniques, unique properties, and potential applications of these particles.

Recent developments have enabled more precise and scalable methods for creating Janus particles, allowing for stringent control over their size, shape, and composition. Controlled surface textures have been shown to significantly enhance the functionality of Janus particles in biomedical applications, such as drug delivery systems, providing controlled and targeted release. These particles have been successfully integrated into smart materials, microfluidic devices, and energy-harvesting technologies, demonstrating their versatility and potential for innovation.

Advances in the controlled surface textures of elastomeric polyurethane Janus particles have significant implications across various fields. In biomedicine, these particles have the potential to revolutionize drug delivery systems and improve the biocompatibility of biomaterials used in implants and prosthetics. In materials science, they can lead to the development of smart materials and responsive sensors capable of adapting to environmental changes. In microfluidics, their surface textures can be used to manipulate the fluid flow and enhance the device performance.

Despite the remarkable progress, several challenges need to be addressed to fully harness the potential of Janus particles. A major challenge is achieving precise control over the size, shape, and composition of Janus particles, which is essential for tailoring their properties and functionalities. Developing scalable and reproducible synthesis methods that allow for precise control of these parameters is crucial. Ensuring the stability and long-term performance of Janus particles, particularly in dynamic environments or under external stimuli, is another critical challenge. Addressing issues related to particle aggregation, degradation, and stability over time is essential for ensuring their reliability and effectiveness in practical applications.

Understanding the complex interactions between Janus particles and their surrounding environments remains a key challenge. This includes elucidating the mechanisms governing particle–particle interactions, as well as interactions with biological systems or other materials. Enhancing our understanding of these interactions will enable more accurate predictions of particle behavior and facilitate the design of tailored particles for specific applications.

Future research on Janus particles is likely to focus on addressing these challenges while exploring new avenues for innovation. This includes the development of novel synthesis techniques, such as bottom-up assembly or microfluidic fabrication methods, to achieve precise control over particle properties. Furthermore, there is growing interest in integrating Janus particles into multifunctional materials and devices, such as responsive coatings, sensors, and drug delivery systems, which will require interdisciplinary collaboration and innovative design strategies.

In summary, while significant advancements have been made in the field of Janus particles, many exciting opportunities for further exploration and development remain. By addressing the existing challenges and embracing new research directions, Janus particles hold great promise for revolutionizing a wide range of applications in materials science, biotechnology, and beyond.
